# Using land‐use history and multiple baselines to determine bird responses to cocoa agroforestry

**DOI:** 10.1111/cobi.13920

**Published:** 2022-06-17

**Authors:** Dominic A. Martin, Estelle Raveloaritiana

**Affiliations:** ^1^ Wyss Academy for Nature University of Bern Bern Switzerland; ^2^ Earth System Science, Department of Geography University of Zurich Zurich Switzerland; ^3^ Plant Biology and Ecology Department University of Antananarivo Antananarivo Madagascar; ^4^ Agroecology, Department of Crop Sciences University of Goettingen Göttingen Germany; ^5^ Sustainable Agricultural Systems and Engineering Laboratory, School of Engineering Westlake University China

**Keywords:** agroecology, cacao, conservation, review, meta‐analysis, forest degradation, forest‐derived agroforest, open‐land‐derived agroforest, ornithology, agrobosque derivado de bosque, agrobosque derivado de campo abierto, agroecología, cacao, conservación, degradación forestal, ornitología, 可可, 保护, 综述, 荟萃分析, 森林退化, 来自森林的农林, 来自开阔地的农林、鸟类学

## Abstract

Agroforests can play an important role in biodiversity conservation in complex landscapes. A key factor distinguishing among agroforests is land‐use history – whether agroforests are established inside forests or on historically forested but currently open lands. The disparity between land‐use histories means the appropriate biodiversity baselines may differ, which should be accounted for when assessing the conservation value of agroforests. Specifically, comparisons between multiple baselines in forest and open land could enrich understanding of species’ responses by contextualizing them. We made such comparisons based on data from a recently published meta‐analysis of the effects of cocoa *(Theobroma cacao)* agroforestry on bird diversity. We regrouped rustic, mixed shade cocoa, and low shade cocoa agroforests, based on land‐use history, into forest‐derived and open‐land‐derived agroforests and compared bird species diversity (species richness, abundance, and Shannon's index values) between forest and open land, which represented the 2 alternative baselines. Bird diversity was similar in forest‐derived agroforests and forests (Hedges’ g* estimate [SE] = ‐0.3144 [0.3416], *p* = 0.36). Open‐land‐derived agroforests were significantly less diverse than forests (g* = 1.4312 [0.6308], *p* = 0.023) and comparable to open lands (g* = ‐0.1529 [0.5035], *p* = 0.76). Our results highlight how land‐use history determined the conservation value of cocoa agroforests. Forest‐derived cocoa agroforests were comparable to the available – usually already degraded – forest baselines, but entail future degradation risks. In contrast, open‐land‐derived cocoa agroforestry may offer restoration opportunities. Our results showed that comparisons among multiple baselines may inform relative contributions of agroforestry systems to bird conservation on a landscape scale.

## Abstract 2

*Article impact statement*: Land‐use history and its comparison with multiple baselines informs assessments of the value of cocoa agroforests for bird conservation.

## Abstract (es)

El historial de uso del suelo y su comparación con diferentes lineas base informan la evaluación del valor de agrobosques de cacao para la coservación de aves

**Resumen:**

Los agrobosques pueden tener un papel importante en la conservación de la biodiversidad dentro de paisajes complejos. Un factor importante que distingue a un agrobosque de otro es el historial de uso del suelo – si el agrobosque está establecido dentro de un bosque o en un área que históricamente fue un bosque y actualmente es un campo abierto. Esta disparidad en el historial del uso del suelo implica que las líneas base de biodiversidad pueden diferir, lo cual debe ser tomado en cuenta cuando se analice el valor de conservación de los agrobosques; específicamente, la contextualización de las comparaciones entre la variedad de líneas base en el bosque y el campo abierto podría enriquecer el entendimiento de la respuesta que tienen las especies. Realizamos dichas comparaciones basadas en datos de un metaanálisis recientemente publicado sobre los efectos de la agrosilvicultura de cacao *(Theobroma cacao)* en la diversidad de aves. Reagrupamos los agrobosques de cacao (rústico, sombra mixta y sombra mínima) en agrobosques derivados del bosque y agrobosques derivados del campo abierto en función al  historial de uso del suelo y comparamos la diversidad de especies de aves (valores de riqueza de especies, abundancia e índice de Shannon) entre bosque y campo abierto, que representaron las dos líneas base alternativas. La diversidad de aves fue similar en los bosques y en agrobosques derivados de ellos (estimado g* de Hedges [SE] = ‐0.3144 [0.3416], *p* = 0.36). Los agrobosques derivados del campo abierto fueron significativamente menos diversos que los bosques (g* = 1.4312 [0.6308], *p* = 0.023) y comparables con los campos abiertos (g* = ‐0.1529 [0.5035], *p* = 0.76). Nuestros resultados destacan cómo el historial de uso del suelo determinó el valor de conservación de los agrobosques de cacao. Los agrobosques de cacao derivados del bosque fueron comparables con las líneas base – generalmente ya degradadas – de bosque disponibles, pero conllevan riesgo de degradación futuro. Al contrario, los agrobosques de cacao derivados del campo abierto podrián ofrecer oportunidades de restauración. Nuestros resultados muestran que las comparaciones entre varias líneas base pueden informar sobre las contribuciones relativas de la agrosilvicultura a la conservación de aves en la escala de paisaje.

## 【摘要】

复合农林可以在复杂景观的生物多样性保护中发挥重要作用。区分复合农林的一个关键因素是土地利用历史, 即复合农林是建立在森林内部还是建立在历史上有森林但目前是开阔地的区域。土地利用历史之间的差异意味着适用的生物多样性基线可能不同, 在评估复合农林的保护价值时应考虑到这一点。具体来说, 考虑环境背景并使用多个基线来比较森林和开阔地, 可以丰富对物种响应的理解。我们根据最近发表的可可 (*Theobroma cacao*) 复合农林业对鸟类多样性影响的荟萃分析数据进行了比较。我们根据土地利用历史, 将乡村、混合遮荫和低遮荫的可可复合农林重新分为来自森林的农林和来自开阔地的农林, 并比较了森林和开阔地之间的鸟类物种多样性 (物种丰富度、丰度和香农指数), 以代表两个替代基线。结果显示, 来自森林的农林和森林中鸟类多样性相似 (Hedges’ g*估计值 [SE] 为−0.3144 [0.3416], *p* = 0.36)。来自开阔地的农林鸟类多样性明显低于森林 (g* = 1.4312 [0.6308], *p* = 0.023), 而与开阔地相当 (g* = −0.1529 [0.5035], *p* = 0.76) 。我们的结果强调了土地利用历史如何决定可可农林的保护价值。来自森林的可可农林与现有的 (通常已退化) 森林基线相当, 但存在未来退化的风险。相比之下, 来自开阔地的可可农林则可能提供恢复的机会。我们的结果表明, 利用多个基线进行比较可以为景观尺度上农林业对鸟类保护的相对贡献提供信息。**【翻译: 胡怡思; 审校: 聂永刚】**

## INTRODUCTION

A careful baseline choice is pivotal for studies on the effect of land‐system change on biodiversity. Such research commonly relies on control‐impact (i.e., space‐for‐time) designs that heavily depend on chosen baselines (i.e., controls) (De Palma et al., 2018). Here, heterogeneous controls can represent a major source of bias (De Palma et al., 2018), and varying controls between studies pose a challenge for synthesis research (Gerstner et al., 2017). To partly address this problem, working with multiple controls can be useful. For example, by comparing vanilla agroforests in Madagascar with little‐used old‐growth forest and heavily used forest fragments, Fulgence et al. (2021) found that amphibian communities in agroforests are significantly less species rich than those in old‐growth forests but comparable to forest fragments; highlighting both opportunities and limitations of amphibian conservation in agroforestry systems.

**FIGURE 1:**
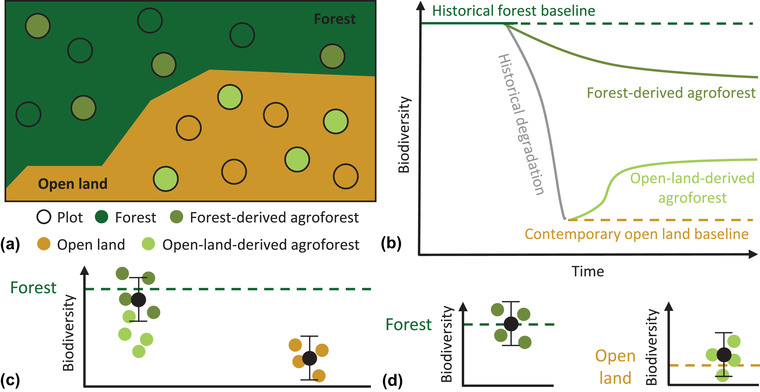
Concept of land‐use history in agroforestry systems. (a) Forest‐derived agroforests established in forests and open‐land‐derived agroforests established on open lands that were historically forested. (b) Hypothetical outcomes of agroforest establishment based on the consideration of land‐use history. Forest‐derived agroforests are likely more biodiverse, but represent a degradation of forest, whereas open‐land‐derived agroforests may increase biodiversity compared to a contemporary open land baseline. (c) Hypothetical relationship of biodiversity with agroforestry without accounting for land‐use history (horizontal line, forest baseline). Forest‐ and open‐land‐derived agroforests are not separated and collectively compared with the forest baseline (horizontal line), as is open land. (d) Hypothetical relationship of biodiversity with agroforestry systems accounting for land‐use history. Forest‐derived agroforests are compared with forests, while open‐land‐derived agroforests are compared with open lands.

In agroforestry research, different baselines – various kinds of forest, perennial monoculture, and open land – are commonly applied (Mupepele et al., 2021), but rarely in combination within the same study (Martin et al., 2020). In this context, considering multiple baselines may be particularly beneficial because agroforests can differ in land‐use history (Martin et al., 2020), meaning they originate from different baselines (forests or open lands) (Fig. 1). A nonquantitative review highlights the importance of land‐use history for ecosystem services and biodiversity in tropical agroforests (Martin et al., 2020). Authors of this article suggest that forest‐derived agroforests typically degrade forests, whereas open‐land‐derived agroforests typically restore open lands. This path‐dependency leads to contrasting outcomes for ecosystem services and biodiversity. Taking the land‐use history of focal agroforests and multiple baselines into account may thus enrich understanding of the value of agroforests for biodiversity and ecosystem services.

One crop commonly farmed in agroforestry systems is cocoa, the most important ingredient of chocolate. Practiced across multiple tropical biodiversity hotspots (FAO, 2020), cocoa agroforestry has value for biodiversity (Bisseleua et al., 2009; Jarrett et al., 2021) and ecosystem services (De Beenhouwer et al., 2013). This value has been recognized in quantitative syntheses on biodiversity (Bennett et al., 2022; Maney et al., 2022) and ecosystem services (Niether et al., 2020) across various types of cocoa agroforestry systems. Nonetheless, cocoa agroforest expansion into forest is a key driver of forest loss in West Africa (Tutu Benefoh et al., 2018) and contributes to forest degradation in Latin America and Southeast Asia (Rice & Greenberg, 2000). But cocoa agroforest can also be established on historically forested open land. For example, on Sulawesi, Indonesia, 50% of cocoa plantations were established on open lands and 50% inside forests (Rice & Greenberg, 2000). Land‐use history may also affect biodiversity (Kessler et al., 2009; Maney et al., 2022), ecosystem services (Nijmeijer et al., 2019), and labor requirements (Ruf, 2001) in cocoa agroforestry systems and might be itself influenced by policy (Orozco‐Aguilar et al., 2021). Importantly, benefits of open‐land‐derived agroforests would likely turn into trade‐offs if agroforests were established on naturally open lands, such as savannas. However, given the climatic niche of cocoa (Schroth et al., 2016), encroachment into forests appears to be a far greater risk than encroachment into naturally open lands (Tutu Benefoh et al., 2018).

In this light, a recent meta‐analysis by Bennett et al. (2022) makes an important contribution to the understanding of bird responses to cocoa agroforestry. Their synthesis brings together data from 23 peer‐reviewed articles in a comparison of rustic cocoa, mixed‐shade cocoa, low‐shade cocoa, and annual monoculture with a forest baseline, thereby combining studies with space‐for‐time designs (De Palma et al., 2018) and a single baseline (i.e., forest). Bennett et al. (2022) compared species richness, abundance, and Shannon's index values before refining their analysis for various functional guilds. The authors also looked at how various habitat features and landscape composition influence bird communities in cocoa agroforests.

We reanalyzed Bennett et al.’s (2022) data to demonstrate how considering land‐use history and multiple baselines enriches understanding of the conservation value of cocoa agroforests for birds.

## METHODS

To separate bird diversity estimates between forest‐ and open‐land‐derived agroforests, we gathered information on the land‐use history of focal agroforests in the introduction and method sections of 16 papers underlying the comparison of 3 bird biodiversity metrics (richness, abundance, Shannon's index values) in the meta‐analysis by Bennett et al. (2022). Additionally, we extracted information on the human influence (e.g., selective logging, secondary vs. primary forest, fragmentation) on forest baselines from the introduction and methods sections of the same studies (Appendix S1). We renamed the land‐use category “annual monoculture” (from Bennett et al. [2022]) as open land, in line with Martin et al. (2020). According to the underlying articles, the open land category includes predominantly annual crops, but also plantain (Harvey & González Villalobos, 2007) and pasture (Estrada et al., 1997; Estrada & Coates‐Estrada, 2005)) (Appendix S1).

The separation based on land‐use history revealed that 10 studies compared forest‐derived agroforests with forests and 4 studies contrasted open‐land‐derived agroforests with forests. Two studies directly compared forest‐ and open‐land‐derived cocoa agroforests (Kessler et al., 2009; Reitsma et al., 2001). We used Bennett et al.’s (2022) data to provide additional results when land‐use history and multiple alternative baselines are considered.

We excluded 2 studies (Schulze et al., 2004; Waltert et al., 2011) in which the same underlying data as in other studies were used (Waltert et al., 2005, 2004) because their use of these data (Bennett et al. 2022) was pseudoreplication (Appendix S1). We also excluded Reitsma et al. (2001) because the study encompasses forest‐ and open‐land‐derived agroforests without separating the 2 during data collection and analysis, preventing the calculation of separate effect sizes. Furthermore, 2 studies took place at the same sites but with different data (Estrada et al., 1997; Estrada & Coates‐Estrada, 2005), 1 of which included only Neotropical migrants (Estrada & Coates‐Estrada, 2005). In this case, we followed Bennett et al. (2022) and included both. We also excluded 3 studies in which diversity measures were not applied to the entire bird community. This left us with 10 studies (Appendix S1).

To directly compare open‐land‐derived agroforests with open lands, we calculated Hedges’ g* for this comparison of 2 effect sizes of different metrics from the same study (Waltert et al., 2004) (Appendix S2). We also calculated Hedges’ g* of effect size for the 2 types of cocoa agroforests and the open lands relative to the available forest baselines. We operationalized this with the same methods and R scripts as in Bennett et al. (2022).

Before fitting Hedges’ g* into a model, we ran a test of the heterogeneity of the data of the full community in the metacont function of R package meta 5.0.2 (Balduzzi et al., 2019). In line with Bennett et al. (2022), we found significant heterogeneity between studies for the comparison of all land systems with forests (Appendix S3). Thus, we built a linear mixed effect model to determine the difference between the 3 land systems (forest‐derived agroforest, open‐land‐derived agroforest, and open land) and forests with the metareg function in the R package metafor 3.0.2 (Viechtbauer, 2010) with the study key as a random effect. We did not find significant heterogeneity for the comparison of open‐land‐derived agroforest with open land (Appendix S3). Therefore, to compare open‐land‐derived agroforests with open land, we used a simple linear model.

## RESULTS

**FIGURE 2:**
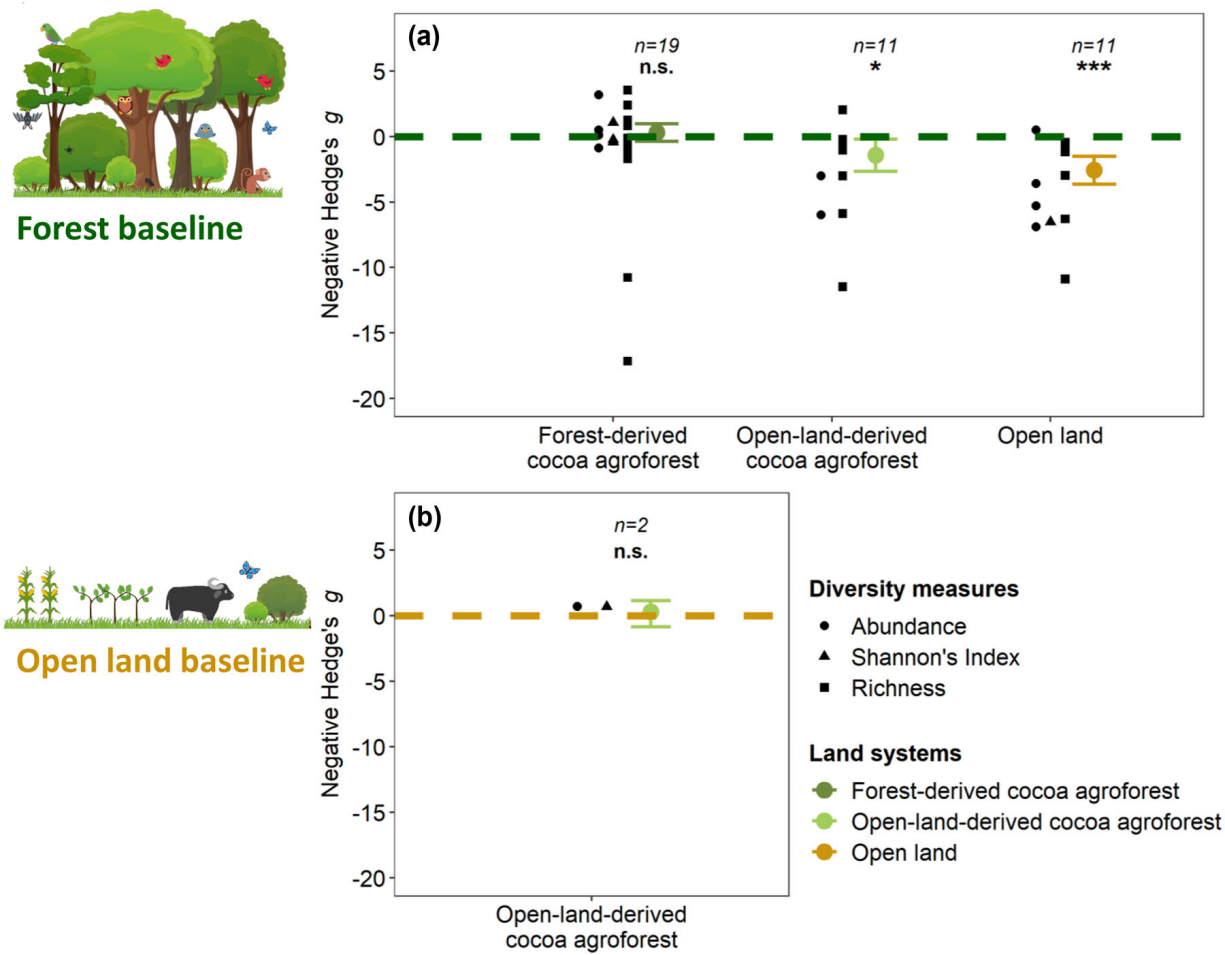
Comparison of (a) forest‐derived cocoa agroforests, open‐land‐derived cocoa agroforests, and open lands with forest baseline (horizontal line) and (b) open‐land‐derived cocoa agroforests compared with open‐land baseline (horizontal line) (asterisks, estimated Hedges' g*: *p< 0.05, ***p< 0.001; n. s., not significant)

Forest‐derived agroforests and the forest baselines hosted a comparable bird diversity (Hedges’ g* estimate [SE] = −0.3144 [0.3416], *p* = 0.36) (Fig. 2a, Appendix S4)) based on 19 diversity measures from seven studies. Open‐land‐derived agroforests had a species diversity comparable to open lands (Hedges’ g* = 0.1529 [0.5035], *p* = 0.76) (Fig. 2b, Appendix S5) based on 2 diversity measures from 1 study. Directly comparing forests‐ and open‐land‐derived agroforests to each other was not possible because only Kessler et al. (2009) included an estimate for forests‐ and open‐land‐derived agroforests. However, when comparing both with the available forest baselines, open‐land‐derived agroforests had significantly lower bird diversity measures than forests (Hedges’ g* = 1.4312 [0.6308], *p* = 0.023) based on 11 diversity measures from 4 studies (Fig. 2a, Appendix S5).

The assessment of forest baselines in underlying studies in Bennett et al. (2022) revealed that only 3 studies compared agroforests with near‐primary forests or mature forests, whereas 13 studies compared agroforests with fragmented, selectively logged, disturbed, used, or secondary forests (Appendix S1).

## DISCUSSION

Our results showed that considering the land‐use history of focal agroforests along with multiple baselines offers an opportunity to draw nuanced conclusions about the bird conservation value of different cocoa agroforestry systems.

Our findings are in line with Bennett et al. (2022) for rustic and mixed‐shade forest‐derived agroforests – these systems hosted a bird diversity comparable to forest baselines (Fig. 2a, Appendix S3). However, the recommendation “implementing rustic and mixed shade agroforestry systems” (Bennett et al. 2022) is controversial because rustic agroforests are by definition forest‐derived (Moguel & Toledo, 1999), so establishing new ones will contribute to forest degradation and associated species turnover – as documented by Bennett et al. (2022). Considering multiple taxa, a recent analysis by Maney et al. (2022) also demonstrates significant decreases in diversity under the conversion of primary forests to forest‐derived cocoa agroforests.

The forest baselines in the articles we analyzed represented fragmented (Faria et al., 2006), disturbed (Davies et al., 2015), partly secondary (Reitsma et al., 2001; Van Bael et al., 2007) or selectively logged forests (Greenler & Ebersole, 2015; Harvey & González Villalobos, 2007) (list of all studies in Appendix S1). Such forests typically have lower bird diversity than less disturbed primary forests – which may themselves lose species (Stouffer et al., 2021) – suggesting shifting baseline syndrome and an overestimated value of forest‐derived agroforests for bird diversity. Nonetheless, we agree with the recommendation of maintaining already established biodiverse forest‐derived agroforests, in line with Martin et al. (2020) and Raveloaritiana et al. (2021).

For low‐shade intensified cocoa, we found that when land‐use history was not considered and the comparison was only with forests, as in Bennett et al. (2022), interpretation challenges resulted that should be considered. All low‐shade intensified agroforests included in Bennett et al. (2022) were established on open lands (Appendix S1). Considering those agroforests as the last step of an intensification from forests via rustic and mixed‐shade cocoa to low‐shade intensified cocoa is thus inaccurate. Instead, these open‐land‐derived low‐shade intensified agroforests could have rehabilitated the open lands on which they were established, leading to possible gains in biodiversity. One study (Waltert et al., 2004) included 2 estimates of bird diversity and data on bird diversity in open lands (i.e., annual cropping in Bennett et al. [2022]), enabling a direct comparison with an alternative baseline. This comparison revealed a diversity in open‐land‐derived agroforests similar to diversity in open lands (Fig. 2b, Appendix S3), but estimates were uncertain given the small sample size. However, Waltert et al. (2004) shows species turnover between the 2 land uses and much lower diversity in cocoa compared with forests, suggesting distinct bird communities in open‐land‐derived agroforests.

The only study included in Bennett et al. (2022) that directly compared forest‐ and open‐land‐derived agroforests (Kessler et al., 2009) shows higher bird diversity in forest‐derived than in open‐land‐derived agroforests, underlining the importance of considering land‐use history. However, Kessler et al. (2009) did not compare their open‐land‐derived agroforests with open lands, prohibiting conclusions on the role of land‐use history. Similarly, Reitsma et al. (2001) mention that focal agroforests differed in land‐use history, but did not consider this difference in their analyses.

Extrapolating to the landscape scale, our results suggest that the benefits of cocoa agroforestry for bird conservation can be best harnessed under the consideration of land‐use history. Seeing open‐land‐derived agroforests as a restoration opportunity (Martin et al. 2020), rather than habitat degradation (Bennett et al., 2022), may help improve management practices so that agroforests deliver for conservation and production goals. For example, this view could help identify historically forested but currently open lands as priority areas for agroforestry systems promotion (Martin et al., 2020) or steer programs to increase shade tree diversity in open‐land‐derived agroforests (Osen et al., 2021), which could benefit birds (Gordon et al., 2007). In contrast, forest‐derived agroforests could serve as buffer zones around protected areas or could be maintained as biodiverse elements within agricultural landscapes (Tscharntke et al., 2011). Evaluating the benefits of agroforestry in response to principal baselines may help make agroforestry a key element of complex agricultural landscapes.

We argue that future analyses and meta‐analyses on biodiversity and ecosystem services in agroforestry systems should consider land‐use history and multiple baselines. Here, going beyond forest and open land as broad categories may offer an interesting research avenue. Specifically, comparing forest‐derived agroforests with old‐growth forests as well as selectively logged or secondary forests could give a more nuanced picture of the value of agroforests for biodiversity, possibly showing that they are less diverse than old‐growth forests but comparable to logged or secondary forests. However, for open land, we were already short on estimates, so a further differentiation in various kinds of open lands would require additional empirical studies in cocoa agroforests.

We conclude that open‐land‐derived cocoa agroforests should not be dismissed simply because they have a lower bird diversity than forest‐derived cocoa agroforests. Rather, by being established on historically forested open lands, they will contribute to agricultural production within working landscapes without worsening the status quo for biodiversity. Moreover, while forest‐derived cocoa agroforests have higher bird diversity, they should not be the preferred form of cocoa production, especially if this entails the further transformation of remaining forests. Considering alternative baselines thus allows for more nuanced policies in the cocoa sector.

## Supporting information

Additional supporting information may be found in the online version of the article at the publisher's website.
