# Supplementary material for: Using land‐use history and multiple baselines to determine bird responses to cocoa agroforestry
Source: Conserv Biol. 2022 Jun 17;36(4):e13920. doi: 10.1111/cobi.13920 (PMC9544578; doi:10.1111/cobi.13920)
Supplement: Supplementary file 1 — Additional supporting information may be found in the online version of the article at the publisher's website. [file COBI-36-0-s001.docx]

# **Supporting information for “Considering land-use history and multiple baselines in a meta-analysis enriches understanding of bird responses to cocoa agroforestry”**

**Appendix S1:** Studies, their location, and the agricultural systems included in the bird diversity meta-analysis (Bennett et al., 2021), as well as their land-use history, the availability of an open-land-baseline, the availability of full community diversity measures, a description of the forest baseline as found in the underlying papers, and comments on pseudo-replication (assessed in this study).

| **Study key (SK)** | **Study** | **Country** | **Agri-cultural system** | **Land-use history** | **Open-land-baseline** | **Full community** | **Forest baseline** | **Com.** |
| --- | --- | --- | --- | --- | --- | --- | --- | --- |
| 451 | Davies et al., 2015 | Solomon Islands | Low & mixed shade cocoa | Open-land-derived | No | yes | Anthropogenically disturbed forest | NA |
| 125 | Faria et al., 2006 | Brazil | Rustic cocoa | Forest-derived | No | yes | Forest fragments | NA |
| 146 | Greenler & Ebersole, 2015 | Costa Rica | Mixed shade cocoa | Open-land-derived | No | yes | Selectively logged remnants | NA |
| 153 | Harvey & González Villalobos, 2007 | Costa Rica | Mixed shade cocoa | Forest-derived | Yes, plantain plantation | yes | Selectively logged | NA |
| 163 | Holbech, 2009 | Ghana | Rustic cocoa | Forest-derived | No | no | Forest remnants | NA |
| 185 | Kessler et al., 2009 | Indonesia | Low, mixed & rustic cocoa | Low: Open-land-derived;  Rustic & mixed: Forest-derived | No | yes | Mature forest with minor rattan extraction | NA |
| 266 | Pardini et al., 2009 | Brazil | Rustic cocoa | Forest-derived | No | no | Forest remnants | NA |
| 440 | Rocha et al., 2019 | Brazil | Rustic cocoa | Forest-derived | No | yes | Mature forest | NA |
| 392 | Van Bael et al., 2007 | Panama | Rustic cocoa | Forest-derived | No | yes | 25% old-growth forest fragments, 75% old secondary forest fragments (both with hunting). | NA |
| 290 | Reitsma et al., 2001 | Costa Rica | Mixed shade cocoa | Forest-derived and open-land-derived | No | excluded for other reason | Selectively logged primary forest (n = 33) and  secondary (n = 67) forest patches. | Excluded due to mixed land-use history |
| 119 | Estrada et al., 1997 | Mexico | Mixed shade cocoa | Forest-derived | Yes, pasture / annual crops | yes | Isolated rain forest fragments | Same site as SK 120 |
| 120 | Estrada & Coates-Estrada, 2005 | Mexico | Mixed shade cocoa | Forest-derived | Yes, pasture / annual crops | no | Isolated rain forest fragments | Same site as SK 119; but only Neotropical Migrants |
| 339 | Schulze et al., 2004 | Indonesia | Low shade cocoa | Open-land-derived | Yes, annual crops | Excluded for other reason | Near-primary forest with rattan extraction and selective logging | Excluded, same data as SK 401 |
| 401 | Waltert et al., 2004 | Indonesia | Low shade cocoa | Open-land-derived | Yes, annual crops | yes | Near-primary forest with rattan extraction and selective logging | Same data as SK 339 |
| 450 | Waltert et al., 2011 | Cameroon | Mixed shade cocoa | Forest-derived | Yes, annual crops | Excluded for other reason | Near-primary forest with very little or no anthr.  activities | Excluded, same data as SK 454 |
| 454 | Waltert et al., 2005 | Cameroon | Mixed shade cocoa | Forest-derived | Yes, annual crops | yes | Near-primary forest with very little or no anthr.  activities | Same data as SK 450 |

**Appendix S2:** Data for two metrics with data on open-land and open-land-derived agroforests used for re-analysis. Abbreviations follow Bennett et al., (2021).

| **STUDY.KEY** | **METRIC** | **N1** | **MEAN1** | **SD1** | **HABITAT1** | **N2** | **MEAN2** | **HABITAT2** | **SD2** |
| --- | --- | --- | --- | --- | --- | --- | --- | --- | --- |
| 401 | ABUND | 4 | 2.75 | 1 | MON | 4 | 3.25 | LNS | 1.5 |
| 401 | RICH | 4 | 7.43 | 2.39 | MON | 4 | 7.35 | LNS | 2.23 |

**Appendix S3.** Test of heterogeneity among effect sizes for the full community for all land-use systems compared to forests and for open-land-derived compared to open-land or monoculture. We used the same threshold as Benett et al., (2021) for the heterogeneity (I.e. I^2 about 75% indicate substantial heterogeneity as do significant values of Q)

| **Community comparisons** | **I^2** | **Q** | **P of Q** |
| --- | --- | --- | --- |
| all land-use systems compared to forest | 95.8 | 953.49 | <0.001 |
| Open-land derived agroforests compared to open land | 0 | 0.14 | 0.7125 |

**Appendix S4:**  Model estimates for the models comparing forest- and open-land-derived cocoa as well as open land to forest. P indicates probability Hedges’ g* estimate does not differ from 0.

| **Independent Variables** | **Hedges' g* estimate** | **SE** | **Lower CI** | **Upper CI** | **P** |
| --- | --- | --- | --- | --- | --- |
| Forest-derived Cocoa | -0.3144 | 0.3416 | -0.9839 | 0.3551 | 0.3574 |
| Open-land-derived Cocoa | 1.4312 | 0.6308 | 0.1949 | 2.6674 | **0.0233** |
| Open land | 2.5716 | 0.5438 | 1.5058 | 3.6374 | **<.0001** |

Mixed-Effects Model (k = 41; tau^2 estimator: DL)

tau^2 (estimated amount of residual heterogeneity): 1.8846 (SE = 0.8203)

I^2 (residual heterogeneity / unaccounted variability): 95.65%

QE(df = 38; Test for Residual Heterogeneity) = 874.3480, p-value < .0001

**Appendix S5:**  Model estimates for the model open-land-derived cocoa to its baseline (open-land). P indicates probability Hedges’ g* estimate does not differ from 0.

| **Independent Variables** | **Hedges' g* estimate** | **SE** | **Lower CI** | **Upper CI** | **P** |
| --- | --- | --- | --- | --- | --- |
| Open-land-derived Cocoa to open land | -0.1529 | 0.5035 | -1.1398 | 0.834 | 0.7614 |

Random-Effects Model (k = 2; tau^2 estimator: DL)

tau^2 (estimated amount of total heterogeneity): 0 (SE = 0.7173)

I^2 (total heterogeneity / total variability): 0.00%

Q(df = 1; Test for Heterogeneity) = 0.1358, p-value = 0.7125

**References**

Bennett, R. E., Sillett, T. S., Rice, R. A., & Marra, P. P. (2021). Impact of cocoa agricultural intensification on bird diversity and community composition. *Conservation Biology*, *n/a*(n/a). https://doi.org/10.1111/cobi.13779

Davies, T. E., Clarke, R. H., Ewen, J. G., Fazey, I. R. A., Pettorelli, N., & Cresswell, W. (2015). The effects of land-use change on the endemic avifauna of Makira, Solomon Islands: Endemics avoid monoculture. *Emu - Austral Ornithology*, *115*(3), 199–213. https://doi.org/10.1071/MU14108

Estrada, A., & Coates-Estrada, R. (2005). Diversity of Neotropical migratory landbird species assemblages in forest fragments and man-made vegetation in Los Tuxtlas, Mexico. *Biodiversity & Conservation*, *14*(7), 1719–1734. https://doi.org/10.1007/s10531-004-0696-x

Estrada, A., Coates-Estrada, R., & Meritt, D. A. (1997). Anthropogenic landscape changes and avian diversity at Los Tuxtlas, Mexico. *Biodiversity & Conservation*, *6*(1), 19–43. https://doi.org/10.1023/A:1018328930981

Faria, D., Laps, R. R., Baumgarten, J., & Cetra, M. (2006). Bat and Bird Assemblages from Forests and Shade Cacao Plantations in Two Contrasting Landscapes in the Atlantic Forest of Southern Bahia, Brazil. *Biodiversity & Conservation*, *15*(2), 587–612. https://doi.org/10.1007/s10531-005-2089-1

Greenler, S. M., & Ebersole, J. J. (2015). Bird communities in tropical agroforestry ecosystems: An underappreciated conservation resource. *Agroforestry Systems*, *89*(4), 691–704. https://doi.org/10.1007/s10457-015-9805-y

Harvey, C. A., & González Villalobos, J. A. (2007). Agroforestry systems conserve species-rich but modified assemblages of tropical birds and bats. *Biodiversity and Conservation*, *16*(8), 2257–2292. https://doi.org/10.1007/s10531-007-9194-2

Holbech, L. H. (2009). The conservation importance of luxuriant tree plantations for lower storey forest birds in south-west Ghana. *Bird Conservation International*, *19*(3), 287–308. https://doi.org/10.1017/S0959270909007126

Kessler, M., Abrahamczyk, S., Bos, M., Buchori, D., Putra, D. D., Gradstein, S. R., Höhn, P., Kluge, J., Orend, F., Pitopang, R., Saleh, S., Schulze, C. H., Sporn, S. G., Steffan-Dewenter, I., Tjitrosoedirdjo, S. S., & Tscharntke, T. (2009). Alpha and beta diversity of plants and animals along a tropical land-use gradient. *Ecological Applications*, *19*(8), 2142–2156. https://doi.org/10.1890/08-1074.1

Pardini, R., Faria, D., Accacio, G. M., Laps, R. R., Mariano-Neto, E., Paciencia, M. L. B., Dixo, M., & Baumgarten, J. (2009). The challenge of maintaining Atlantic forest biodiversity: A multi-taxa conservation assessment of specialist and generalist species in an agro-forestry mosaic in southern Bahia. *Biological Conservation*, *142*(6), 1178–1190. https://doi.org/10.1016/j.biocon.2009.02.010

Reitsma, R., Parrish, J. D., & McLarney, W. (2001). The role of cacao plantations in maintaining forest avian diversity in southeastern Costa Rica. *Agroforestry Systems*, *53*(2), 185–193. https://doi.org/10.1023/A:1013328621106

Rocha, J., Laps, R. R., Machado, C. G., & Campiolo, S. (2019). The conservation value of cacao agroforestry for bird functional diversity in tropical agricultural landscapes. *Ecology and Evolution*, *9*(14), 7903–7913. https://doi.org/10.1002/ece3.5021

Schulze, C. H., Waltert, M., Kessler, P. J. A., Pitopang, R., Veddeler, D., Mühlenberg, M., Gradstein, S. R., Leuschner, C., Steffan-Dewenter, I., & Tscharntke, T. (2004). Biodiversity Indicator Groups of Tropical Land-Use Systems: Comparing Plants, Birds, and Insects. *Ecological Applications*, *14*(5), 1321–1333.

Van Bael, S. A., Bichier, P., Ochoa, I., & Greenberg, R. (2007). Bird diversity in cacao farms and forest fragments of western Panama. *Biodiversity and Conservation*, *16*(8), 2245–2256. https://doi.org/10.1007/s10531-007-9193-3

Waltert, M., Bobo, K. S., Kaupa, S., Montoya, M. L., Nsanyi, M. S., & Fermon, H. (2011). Assessing Conservation Values: Biodiversity and Endemicity in Tropical Land Use Systems. *PLoS ONE*, *6*(1), e16238. https://doi.org/10.1371/journal.pone.0016238

Waltert, M., Bobo, K. S., Sainge, N. M., Fermon, H., & Mühlenberg, M. (2005). FROM FOREST TO FARMLAND: HABITAT EFFECTS ON AFROTROPICAL FOREST BIRD DIVERSITY. *Ecological Applications*, *15*(4), 1351–1366. https://doi.org/10.1890/04-1002

Waltert, M., Mardiastuti, A., & Mühlenberg, M. (2004). Effects of Land Use on Bird Species Richness in Sulawesi, Indonesia. *Conservation Biology*, *18*(5), 1339–1346. https://doi.org/10.1111/j.1523-1739.2004.00127.x
